# Network Pharmacological Analysis on the Herbal Combinations for Mitigating Inflammation in Respiratory Tracts and Experimental Evaluation

**DOI:** 10.3390/healthcare11010143

**Published:** 2023-01-03

**Authors:** Dongyeop Jang, Myong Jin Lee, Kang Sub Kim, Chang-Eop Kim, Jong Ho Jung, Minkwan Cho, Bo-Hee Hong, Shin Jung Park, Ki Sung Kang

**Affiliations:** 1 College of Korean Medicine, Gachon University, Seongnam 13120, Republic of Korea; 2 Chong Kun Dang (CKD) Pharm Research Institute, Yongin 16995, Republic of Korea

**Keywords:** inflammation, COVID-19 complications, herbal medicine, synergistic effects, network pharmacology, design of experiments

## Abstract

The regulation of inflammatory mediators, such as TNF-α, IL-6, IL-1β, and leukotriene B4, could play a crucial role in suppressing inflammatory diseases such as COVID-19. In this study, we investigated the potential mechanisms of drug combinations comprising Ephedrae Herba, Schisandra Fructus, Platycodonis Radix, and Ginseng Radix; validated the anti-inflammatory effects of these drugs; and determined the optimal dose of the drug combinations. By constructing a herb-compound-target network, associations were identified between the herbs and tissues (such as bronchial epithelial cells and lung) and pathways (such as the TNF, NF-κB, and calcium signaling pathways). The drug combinations exerted anti-inflammatory effects in the RAW264.7 cell line treated with lipopolysaccharide by inhibiting the production of nitric oxide and inflammatory mediators, including TNF-α, IL-6, IL-1β, and leukotriene B4. Notably, the drug combinations inhibited PMA-induced *MUC5AC* mRNA expression in NCI-H292 cells. A design space analysis was carried out to determine the optimal herbal medicine combinations using the design of experiments and synergy score calculation. Consequently, a combination study of the herbal preparations confirmed their mitigating effect on inflammation in COVID-19.

## 1. Introduction

The COVID-19 pandemic is a global outbreak of coronavirus which is caused by severe acute respiratory syndrome coronavirus 2 (SARS-CoV-2). Through life-threatening systemic inflammatory syndromes like COVID-19, a sudden acute elevation of circulating levels of different cytokines and immune-cell hyperactivation generally promote the cytokine storm which can be triggered by various injurious stimuli, such as pathogens and damaged tissues [1]. A common characteristic for a lot of patients who get severe COVID-19 is critical lung failure by an excessively dynamic immune response, overproducing many inflammatory cytokines and chemokines. This high-level release of cytokines regulates immune responses to inflammation and infection through complicated interactions [2]. The onset of the immune system is induced by pro-inflammatory cytokines such as TNF-α, IL-6, and IL-1β involving the activation of the immune cells such as macrophages, lymphocytes, and neutrophils as regulators of the immune response [3]. Leukotriene B4 is one of the pro-inflammatory mediators that induce the accumulation of neutrophils, monocytes, and eosinophils, stimulating the recruitment of numerous pro-inflammatory cytokines and mediators [4]. Coronavirus disease 2019 (COVID-19) caused a global pandemic that has led to numerous deaths. The clinical symptoms of COVID-19 include fever, cough, and respiratory disorders [5]. Since COVID-19, an RNA virus, frequently undergoes mutation, a specific drug is not available to treat the disease.

Many patients who had COVID-19 lasted for several months and they had systemic (fatigue, poor concentration, chronic malaise), respiratory (dyspnea, persistent cough), neuropsychiatric (insomnia, chronic headache, brain fog), and cardiac manifestations [6]. Inflammatory markers and cytokines were found to increase in the blood of patients with COVID-19 [7]. Further, the increase in leukotriene is maintained for 5 months, even after recovery from COVID-19, thereby increasing the sensitivity of respiratory inflammation and resulting in complications [8]. The underlying mechanisms for long-term symptoms are elusive at present. Therefore, we hypothesize that inflammatory condition persists in the long-term following COVID-19 infection and it’s related to hyperinflammation. Traditional Chinese medicine has struggled to treat infectious diseases for almost 2000 years [9], and has been considered to have not only fewer adverse effects than other drugs for infections but is also effective [10]. In our ongoing project on the quality evaluation of various herbal medicine prescriptions, we found that herbal combinations can help mitigate inflammation status in post-COVID-19 syndrome based on several screening results. In this study, we attempted to determine the anti-inflammatory effect of an optimal combination comprising Ephedrae Herba (EH), Schisandrae Fructus (SF), Platycodonis Radix (PR), and Ginseng Radix (GR).

Historically, traditional Chinese medicine (TCM) has been developed to treat and prevent epidemic diseases [11]. Drugs in TCM have been shown that they may alleviate symptoms in respiratory diseases [10,12,13] and regulate cytokines [13,14,15]. These studies suggest that drugs in TCM can be potential drugs for mitigating post-COVID-19 syndromes. More specifically, EH, SF, PR, and GR are considered to treat respiratory diseases. EH has been used to treat respiratory symptoms, including cough, headache, pain, and high fever. For example, EH is the main component of Maoto, a formula used in traditional Asian medicines to treat influenza-like symptoms [16]. Drug combinations comprising EH inhibited respiratory syncytial virus infectivity in A549 cell lines [17] and reduced airway damage in asthmatic rats [18]. SF has been prescribed for chronic cough in traditional Chinese medicine [19]. SF displayed antitussive activity in guinea pigs exposed to cigarette smoke [19]. Schisandrin B inhibited the pro-inflammatory factors associated with asthma and regulated the NF-kB signaling pathway [20]. PR has been considered a basic herbal medicine for clearing heat and nourishing yin in acute and chronic inflammation and upper respiratory tract infections [21,22]. Platycodins, the main components in PR, suppressed the expression of PMA-induced *MUC5AC* in the NCI-H292 cell line, mucin secretion from the trachea in rat models [23], and TNF-α expression at the transcriptional level in NCI-H292 cells [24]. GR is one of the most famous herbs used in traditional Chinese medicine to enhance the qi of the spleen and lung [25]. GR regulates expressions of proinflammatory cytokines (including TNF-α, IFN-γ, and interleukins) and chemokines in epithelial cells and macrophages in the airway. Interestingly, the modified Ginseng-Schisandra decoction improves clinical symptoms and serum immunoglobulins in children with spleen deficiency syndrome following recurrent respiratory tract infection [26]. These findings suggest that the synergistic use of EH, SF, PR, and GR can ameliorate inflammatory diseases and symptoms following virus infection of the respiratory system.

The clinical relevance of drug synergism for disease control and prevention has been widely acknowledged [27]. For traditional Chinese herbal medicine, the synergistic use of herbs in formulas is particularly important [28]. For example, theories such as “couplet medicine” and “sovereign, minister, assistant, and courier” are considered during the herbal combination. According to these theories, herbal combinations increase the therapeutic effects of herbs. To elucidate the synergistic interactions between the herbs, methods to quantify synergy between drugs, such as combination index, isobole method, and systems biology, are applied to formulas or herbal pairs in traditional Chinese herbal medicine [28].

Using network pharmacological analysis, the system-level mechanisms of drugs composed of multiple compounds, including natural products, have been elucidated [25,29,30]. For COVID-19, the network pharmacological analysis of traditional Chinese herbal drugs has been performed [31,32,33,34]. However, these analyses were limited as they lack biological evidence indicating the therapeutic effects of the drugs, although the new indications of the drugs have not been generally accepted. In our study, we analyzed the biological mechanisms of EH, SF, PR, and GR on inflammatory pathways and confirmed the anti-inflammatory effects of drug combinations to verify their possible mitigating effects on inflammation in respiratory tracts focusing on COVID-19.

## 2. Materials and Methods

### 2.1. Network Pharmacological Analysis

Network pharmacological analysis in TCM focuses on the interaction between multiple compounds in drugs and their multiple targets to elucidate system-level effects and mechanisms of drugs [35]. In this study, TCMSP (https://tcmsp-e.com/tcmsp.php (accessed on 30 May 2022)) [36] was employed to collect compound information on EH, PR, GR, and SF. TCMSP collects information about compounds of herbs by conducting a comprehensive literature search and by retrieving structure data about compounds from PubChem [37], ChEMBL [38], and ChemSpider [39]. To predict drug-like compounds in herbal drug, we applied a quantitative estimate of drug-likeness (QED), which infers drug-likeness based on molecular properties (such as octanol–water partition coefficient and number of hydrogen bond donors) and outperform Lipinski’s rule of five in distinguishing drugs from a set of chemicals [40]. Based on QED, compounds with a low possibility of exerting therapeutic effects via oral administration were excluded. The threshold value of QED for excluding the compounds was 0.35 [40]. The STITCH database (http://stitch.embl.de/ (accessed on 30 May 2022) was used to identify the potential protein targets of compounds [41]. For a given chemical-protein interaction, the STITCH database provides four types of individual scores indicating the likelihood or relevance of each individual type of evidence: experimental evidence of direct chemical-protein bindings, text mining of MEDLINE and OMIM, annotated chemical-protein interactions from other databases, and 2D chemical fingerprint-based prediction [42]. The individual scores are combined into an overall score (called the combined score), and the closer the score is to 1, the greater the likelihood of interaction. In this study, highly confident interactions with combined scores greater than 0.7 were selected.

We first estimate the relationship between the drug and COVID-19 by conducting a enrichment analysis that statistically analyzes overlaps between potential targets and COVID-19-related genes obtained from the CTD database [43] and KEGG [44]. Next, to determine whether the potential targets are related to tissues or pathways related to COVID-19, enrichment analysis was conducted using information about tissue-elevated gene expression in The Human Protein Atlas [45] and information about pathway-related genes in the KEGG [44]. Enrichr, an open-source enrichment analysis tool [46], was used for enrichment analysis, and adjusted *p*-values and combined scores (the logarithm of the multiplication of the *p*-value and z-score of overlap between targets and gene sets) were obtained.

### 2.2. Preparation of MHRCs

In this study, soft extracts or concentrates prepared according to pharmaceutical standards were used. EH, PR and SF were purchased from Hanpoong Pharm (HANPOONG PHARM & FOODS, Seoul, Korea). GR was obtained from Bolak (Hwaseong, Korea).

Table 1 shows the combined doses of the herbal extract used to confirm the synergistic effect in this study.

### 2.3. Cell Culture and Nitrite Assay

The murine RAW264.7 macrophage cell line, obtained from Korea Cell Line Bank (Seoul, Republic of Korea), was cultured in Dulbecco’s modified Eagle’s medium (DMEM; Corning, Manassas, VA, USA) supplemented with 10% heat-inactivated fetal bovine serum (Atlas Biologicals, Fort Collins, CO, USA) and 1% antibiotics (100 U/mL of penicillin and 100 U/mL of streptomycin; Gibco BRL, Carlsbad, MD, USA) at 37 °C in a 5% CO_2_ humidified incubator. The cells were seeded at a density of 5 × 10^4^ cells/well in a 96-well microplate. Thereafter, the cells were pretreated with different doses of the combinations of EH, PR, GR, and SF for 2 h and then induced with 100 ng/mL LPS (Sigma Aldrich Inc., St. Louis, MO, USA). After 22 h, the cell culture medium was mixed with an equal volume of Griess reagents composed 2% sulfanilamide (Sigma Aldrich Inc., St. Louis, MO, USA) and naphthylethylenediamine dihydrochloride (Sigma Aldrich Inc., St. Louis, MO, USA) in 5% phosphoric acid (Sigma Aldrich Inc., St. Louis, MO, USA), and the absorbance was measured at 540 nm using a microplate reader (Bio-Tek Instruments, Winooski, VT, USA).

### 2.4. NCI-H292 Cell Culture and PMA-Induced MUC5AC mRNA Expression

NCI-H292 human mucoepidermoid pulmonary carcinoma cells (Korean Cell Line Bank, Seoul, Korea) were grown in RPMI-1640 (Corning, Manassas, VA, USA) containing 10% fetal bovine serum (FBS; Atlas, Fort Collins, CO, USA) and 100 U/mL of penicillin–streptomycin (Gibco, Grand Island, NY, USA) in a humid atmosphere containing 5% CO_2_ at 37 °C. The cells were seeded in 6-well plates at a density of 5 *×* 10^5^ cells/well. After starving in serum-free RPMI-1640, the cells were treated with 10 nM of dexamethasone or 100 µg/mL of other MHRCs for 1 h and subsequently exposed to 100 nM PMA for 24 h. Total cellular RNA was isolated using the RNeasy Mini Kit (Qiagen, Germantown, MD, USA). RNA was reverse-transcribed into cDNA using the RevertAid First Strand cDNA Synthesis kit (Thermo Fisher Scientific, Eugene, OR, USA). Polymerase chain reaction (PCR) was performed using the AccuPower^®^ 2X GreenStar™ qPCR Master Mix (Bioneer, Daejeon, Republic of Korea). The sense and antisense primers are listed in Table 2. β-actin was selected as the internal control. The amplification conditions were as follows: 50 °C for 2 min; 95 °C for 10 min; followed by 40 cycles of 95 °C for 15 s, 60 °C for 1 min, and 95 °C for 15 s; 60 °C for 1 min; 95 °C for 15 s. The Quant Studio 3 real-time PCR system (Applied Biosystems, Foster City, CA, USA) was used.

### 2.5. Enzyme-Linked Immunosorbent Assay (ELISA)

RAW264.7 cells were seeded in 6-well plates (5 × 10^5^ cells/well). After incubation, macrophages were treated with 1 mg/mL of the EH, PR, GR, and SF combination followed by LPS for an additional 22 h. The culture media were collected, and the concentrations of TNF-α, IL-6, and IL-1β (BD Biosciences, San Diego, CA, USA) and that of leukotriene B4 (Caymanchem, Ann Arbor, MI, USA) in the supernatants were determined using Enzyme-linked immunosorbent assay (ELISA) kits, according to the manufacturer’s instructions. The absorbance was measured at 540 nm using a spectrophotometer.

### 2.6. Synergy Scores

To quantitatively measure drug interactions, the HSA synergy score $S_{HSA}$ [47] and Bliss synergy score $S_{Bliss}$ [48] were calculated for each combination of drugs as follows:

(1) $$S_{HSA}=y_{c}-\max\left(y_{1}\left(x_{1}\right),\;y_{2}\left(x_{2}\right)\right)$$

(2) $$\;S_{Bliss}=y_{c}-\left(y_{1}\left(x_{1}\right)+y_{2}\left(x_{2}\right)-y_{1}\left(x_{1}\right),\;y_{2}\left(x_{2}\right)\right)$$

where $y_{c}$ is the observed effect of the drug combination and $y_{n}\left(x_{n}\right)$ is the effect of a single drug n at the concentration $x_{n}$. A difference was found between the HSA score and Bliss score in an assumption of drug synergism., i.e., when there is no synergism. HSA score assumes synergism is absent when the effects of a drug combination do not surpass that of each drug in the combination [47]. In contrast, the Bliss score assumes synergism when the effects of a drug combination do not surpass the effects of drugs when each drug in the combination acts “independently” [48]. Note that a drug combination with an HSA score greater than 0 can have a Bliss score less than 0.

### 2.7. Design of Experiment (DOE) and Design Space (DS)

DOE aims to explore optimized multiple factors that influence responses with the limited number of experiments. In this study, the DOE proceeds in the following order:

1. Evaluation of the impact between factors and response value; This identifies whether factors and response correlate with each other, and finds out the tendency.
2. Identification of major factors affecting response value; The major factors determining the responses are identified through this step and it confirms the degree of influence.
3. Creation of a statistical model for the factors and response correlations; This creates an alternative model that approximates the correlation between factors and response.
4. An optimal design solution to satisfy responses; The combination of factors is found that obtains the most desirable responses.

DS, also known as a statistical space, is derived by the followed processes. The DS indicates the range of factors in which the responses satisfy criteria for individual responses which are set in the study (the estimation of DS is performed by the equation according to Section 2.8) Operating space (OS) indicates feasible ranges of all factors derived by the intersection of DS.

In other words, an estimated function model that approximates the correlation between factors (doses of GR and SF) and responses (NO, TNF-α, IL-6, IL-1β, and LTB4) is generated through DOE. DS which is the level of factors C and D that satisfies the target range of each response (NO, TNF-α, IL-6, IL-1β, and LTB4) is derived. Finally, OS which is a more feasible area within DS is derived.

### 2.8. Estimated Regression Equations

The estimated regression equation is derived using the DOE method. Factorial design (FD), which is one of the DOE methods, appears as a linear regression equation. The response surface methodology (RSM), another method, appears as a quadratic regression equation. RSM utilizes polynomial regression of the quadratic model to determine the precise and optimal factor combinations for target responses. The FD and RSM of DOE are represented by Equations (3) and (4):

(3) $$y(x_{1},\;x_{2},\;\ldots ,x_{m})=\beta_{0}+\overset{m}{\underset{j=1}{\sum }}\beta_{j}x_{j}+\overset{m-1}{\underset{i=1}{\sum }}\overset{m}{\underset{j=2}{\sum }}\beta_{ij}x_{i}x_{j},\;(i<j)$$

(4) $$y(x_{1},\;x_{2},\;\ldots ,x_{m})=\beta_{0}+\overset{m}{\underset{j=1}{\sum }}\beta_{j}x_{j}+\overset{m-1}{\underset{i=1}{\sum }}\overset{m}{\underset{j=2}{\sum }}\beta_{ij}x_{i}x_{j}+\overset{m}{\underset{j=1}{\sum }}\beta_{jj}x_{j}^{2},\;(i<j)$$

where $\beta_{0}$, $\beta_{j}$, $\beta_{ij}$, and $\beta_{jj}$ are regression coefficients of the constant, linear, quadratic, and second-order model, respectively; $x_{i}$ and $x_{j}$ are input variables in the regression function; and $y(x_{1},\;x_{2},\;\ldots ,x_{m})$ is the response. DS is determined based on an estimated regression equation established.

## 3. Results

This study was conducted to identify the optimal combination of drugs by three-steps; First, network pharmacological analysis was conducted to derive each herb that would show anti-inflammatory effects on COVID-19 (Section 3.1 and Section 3.2); Second, in vitro experiments were performed to confirm the anti-inflammatory effects and measure the synergistic effects of combinations of different ratios of drugs (Section 3.3, Section 3.4 and Section 3.5); Third, DOE analysis based on data from the in vitro experiments was applied to optimize the ratio of drugs in the drug combination for maximizing anti-inflammatory effects (Section 3.6).

### 3.1. Association of the Drugs and COVID-19

**Figure 1:**
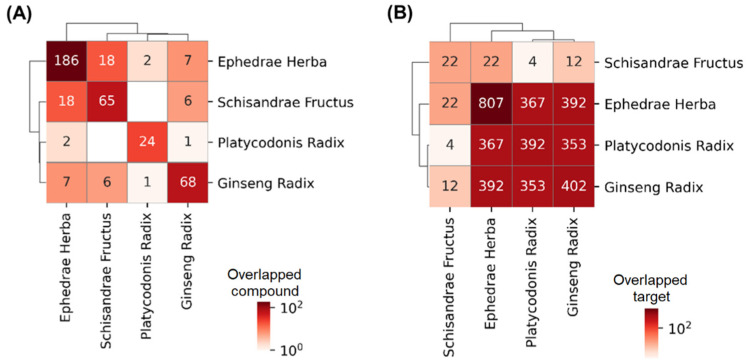
Overlaps in the bioactive compounds or the targets of herbs. The number and color in each cell indicate the number of overlapped (**A**) compounds or (**B**) targets of the herbs corresponding to the row and column (The numbers and colors on the diagonal line indicate the number of compounds and targets corresponding to the herb).

We evaluated the bioactive compounds in herbs that make up the drug combination. EH, PR, GR, and SF were found to have 186, 24, 68, and 65 bioactive compounds, respectively (Figure 1A), and 807, 392, 402, and 22 protein targets (Figure 1B), respectively. Although the herbs tended to have distinct bioactive compounds, it was shown that the targets of herbs have a tendency to be overlapped.

**Figure 2:**
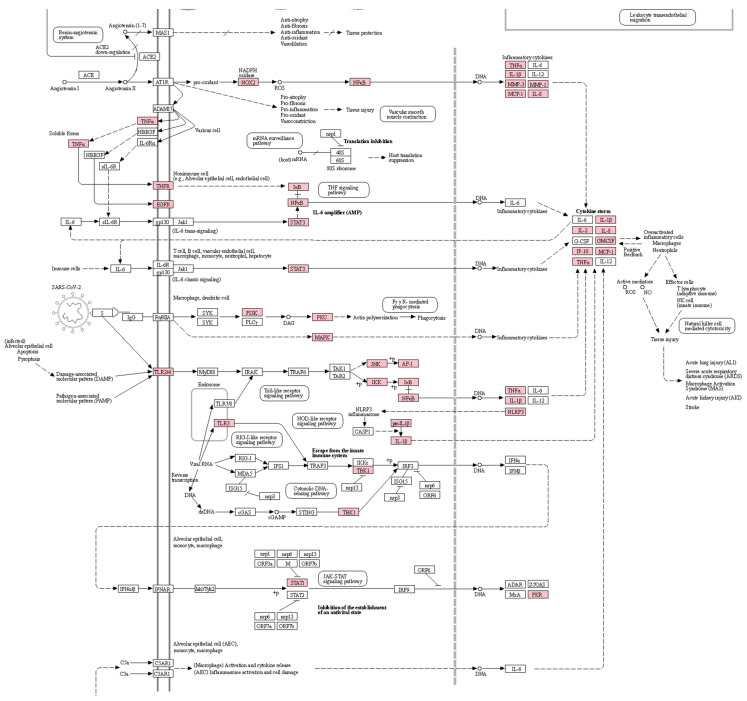
A pathway for coronavirus diseases highlighting the potential targets of the drug combination. Boxes and arrows between them indicate the proteins that consist of the pathway and interactions between the proteins, respectively. Pink-colored boxes indicate the targets of the drug combination. The graph image was generated by a KEGG mapper and cropped by the authors because of the page limit. The uncropped image is shown in Supplementary Figure S1.

We first estimated the relationship between COVID-19 and the drugs by enrichment analysis for COVID-19-related genes and potential targets of the drug. It was shown that targets of the drug combinations (including TNF, IL1B, IL10, and CCL2) (*p* $=5.72\times 10^{-5}$) and, specifically, its individual drugs including EH (*p* = 0.00142), PR (*p* $=4.37\times 10^{-4}$), and GR (*p* $=5.81\times 10^{-5}$) are significantly overlapped with COVID-19-related genes obtained from the CTD database. Moreover, targets of drug combination (*p* $=8.11\times 10^{-12}$), EH (*p* $=4.64\times 10^{-11}$), PR (*p* $=1.14\times 10^{-7}$), and GR (*p* $=1.61\times 10^{-7}$) were also shown to be significantly overlapped with genes in the pathway of coronavirus diseases that are independently collected by KEGG (Table 3). The overlapped targets are especially associated with the signaling of inflammatory cytokines including TNF-α, interleukins, and NF-κB resulting in cytokine storms (Figure 2), suggesting that the mechanisms of these drugs for COVID-19 and its postinfectious syndromes are associated with regulation of inflammatory signaling. Therefore, we will explore the effects and mechanisms of the drugs focusing on the regulation of inflammation in respiratory systems.

### 3.2. Enrichment Analysis for COVID-19-Related Tissues and Pathways

The relationship between the drugs and tissues related to COVID-19 was determined by performing enrichment analysis to estimate the degree of overlap of the drug targets with elevated genes in tissues. The targets of the drug combination showed significant overlaps with genes that were elevated in expression in bronchial epithelial cells (*p* $=5.67\times 10^{-5}$) and lung (*p* = 0.047), implying that the drug combination is related to these tissue types. However, at the single herb level, only the targets of EH significantly overlapped with elevated genes in bronchial epithelial cells (*p* = $2.40\times 10^{-5}$) (Table 4). This finding implies that the drug combination can act on the tissues via complementary interactions among the individual drugs.

To decipher the potential mechanisms of the drug combinations in COVID-19, enrichment analysis was performed to determine the impact of the targets on the Kyoto Encyclopedia of Genes and Genomes (KEGG) pathways related to TNF-α, IL-1β, and BLT1, which are inflammatory mediators associated with inflammation in the upper respiratory tract. The targets of the drug combination were found to overlap significantly with the proteins of the TNF-α (*p* = $2.68\times 10^{-21}$), NF-κB (*p* = $1.13\times 10^{-10}),$ and calcium (*p* = $7.96\times 10^{-34}$) signaling pathways. In particular, significant overlapping was found between the targets of EH, PR, and GR (except those of SF) and the proteins of these pathways (Table 5). This finding implies that the drug combination can affect the TNF-α, NF- κB, and calcium signaling pathways via the overlapped protein targets.

**Figure 3:**
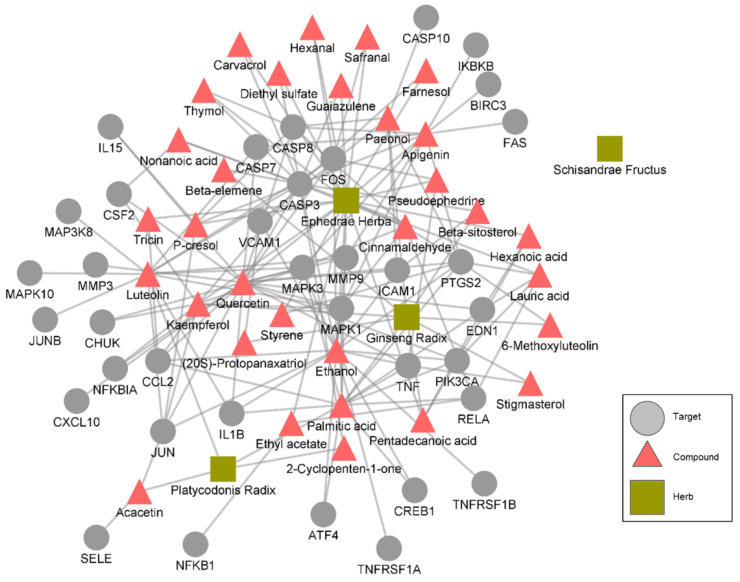
A herb-compound-target network that is specific to the TNF signaling pathway. The targets that represent the “target” nodes are proteins related to the TNF signaling pathway.

The mechanism whereby the herbs act on these pathways was determined by assessing the bioactive compounds in the herbs whose targets are related to the pathways. EH, PR, GR, and SF were found to have 30, 4, 12, and 1 bioactive compound, respectively, related to the pathways (Table 6). The targets of the bioactive compounds related to the TNF, NF-κB, and calcium signaling pathways are shown in Figure 3. In case of the TNF signaling pathway, compounds in EH, GR, and PR interact with proteins related to TNF signaling. Specifically, stigmasterol in GR, pseudoephedrine and quercetin in EH were found to be related with TNF, and multiple compounds are related with apoptosis (via CASP3, 7, and 10), MAPK signaling pathway (via proteins including MAPK1 and MAPK3), and NF-kappa B signaling pathway (via proteins including NFKBIA) that are involved in TNF signaling (Figure 3). These results imply that the drug combination exhibits anti-inflammatory effects via the comprehensive interactions among the bioactive compounds of the herbs in the drug combination.

### 3.3. Effects of the Drug Combinations on LPS-Induced Inflammation in RAW264.7 Cells and Their Synergistic Score

**Figure 4:**
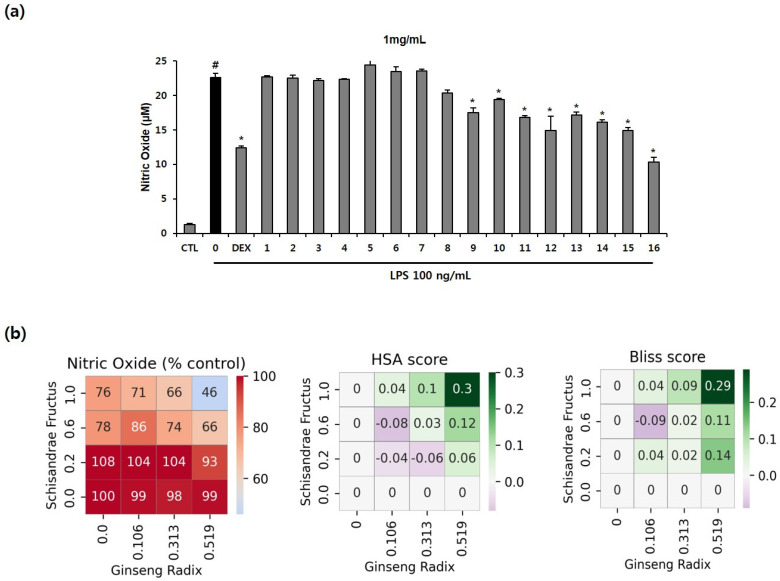
Anti-inflammatory effects of drug combinations 1–16 and their synergistic scores in RAW264.7 macrophages. (**a**) The cells were treated with 1 mg/mL of the combinations for 2 h, followed by LPS (100 ng/mL) for 22 h. The level of NO in the culture supernatants was determined using the Griess method. The numbers on the *x*-axis indicate the codes given for the individual combinations (Table 1). The values are presented as mean ± SD (n = 3). # *p* < 0.05 compared to the untreated group. * *p* < 0.05 compared with the LPS-treated group. (**b**) Percent of the test group relative to the negative control, HSA score, and Bliss score for NO concentration. The *x*-axis and *y*-axis indicate the dose of drug C or drug D in each cell, and each cell represents one drug combination formulated with the dose of the drug corresponding to the *x*- and *y*-axis. The red color indicates a value higher than that of the positive control (0.544).

We evaluated the production of NO with the combinations of EH, PR, GR, and SF in LPS-induced inflammation in RAW264.7 cells. Further, we determined the cytotoxic effects of various concentrations of the EH, PR, GR, and SF combinations in RAW264.7 cells. Most combinations were cytotoxic up to 3 mg/mL (Supplementary Figure S2). NO production was tested with 1 mg/mL of the combinations that exerted not toxic effect in LPS-stimulated murine macrophages. As shown in Figure 4a, only the nitrite level of the LPS-treated group increased relative to that of the untreated group. Combinations 1-8 did not affect the release of NO, while combinations 9–16 inhibited nitrite production compared to LPS treatment. In particular, combination 16 (SF: 0.519 g, GR: 1.0 g) induced a greater reduction in NO level than dexamethasone, which was employed as the positive control (Figure 4a).

Synergy scores were calculated to determine the dose of the drug combination that induces synergy. Combination 16 (SF: 0.519 g, GR: 1.0 g) induced the highest level of inhibition of all combinations and had the highest bliss score and HSA score, indicating that this combination exerts synergistic therapeutic effects. Combination 16 also exhibited a higher anti-inflammatory effect than the positive control (Figure 4b). Overall, these results suggest that combination 16 could exhibit synergy and may serve as an optimal drug combination with maximal anti-inflammatory effects.

### 3.4. Effects of the Drug Combinations on LPS-Mediated Pro-Inflammatory Cytokine Production

**Figure 5:**
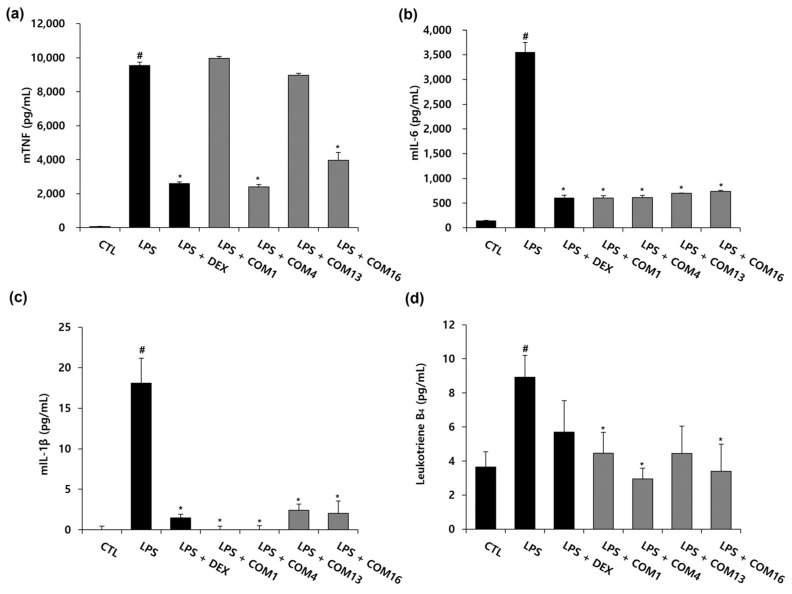
Effects of the drug combinations on the LPS-induced production of inflammatory cytokines in RAW264.7 cells. Murine macrophages were pretreated with 1 mg/mL of the drug combination 1, 4, 13, and 16 (drug combination 1 (COM1), EH: 0.273 g and PR: 0.263 g; drug combination 4 (COM4), EH: 0.273 g, PR: 0.263 g and SF: 0.519 g; drug combination 13 (COM13), EH: 0.273 g, PR: 0.263 g and GR: 1.0 g; drug combination 16 (COM16), EH: 0.273 g, PR: 0.263 g, SF: 0.519 g and GR: 1.0 g) for 2 h, followed by 100 ng/mL of LPS for 22 h. Cytokine accumulations for TNF (**a**), IL-6 (**b**), IL-1β (**c**) and Leukotriene B4 (**d**) were analyzed by an ELISA at 540 nm. The values are presented as mean ± SD (n = 3). # *p* < 0.05 compared to the untreated group. * *p* < 0.05 compared with the LPS-treated group.

As shown in Figure 4, the combination 16 showed the highest synergistic effects on NO inhibition (Bliss score: 0.29, HSA score: 0.3). After NO inhibition experiments, to explore synergistic effects (between SF and GR in combination 16) on the production of TNF-α, IL-6, IL-1β, and leukotriene B_4_ in LPS-activated murine macrophages, ELISA was performed. Combination 1 (without SF and GR), 4 (without GR), and 13 (without SF) were selected by control groups that exclude GR or SF from combination 16. As depicted in Figure 5, all treatments comprising 1 mg/L of the SF and GR drug combinations (1, SF: 0 g and GR: 0 g; 4, SF: 0.519 g and GR: 0 g; 13, SF: 0 g and GR: 1.0 g; 16, SF: 0.519 g and GR: 1.0 g) inhibited the production of IL-6 and IL-1β completely compared to LPS treatment alone. The production of the cytokines IL-1β and IL-6 were completely inhibited in the drug treatment groups compared to the LPS group. The release of TNF was suppressed by combinations 4 and 16 to a similar level of suppression induced by dexamethasone. The secretion of leukotriene B_4_ was reduced to a greater extent by all combinations relative to the positive control (Figure 5). Our data indicate that the inhibition of cytokine production by combination 16 (SF: 0.519 g, GR: 1.0 g) was consistent with that observed in the nitrite assay of LPS-treated RAW 264.7 cells.

### 3.5. Effect of the Multi-Herb Ratio Combinations (MHRCs) on PMA-Induced MUC5AC mRNA Expression in NCI-H292 Cells

**Figure 6:**
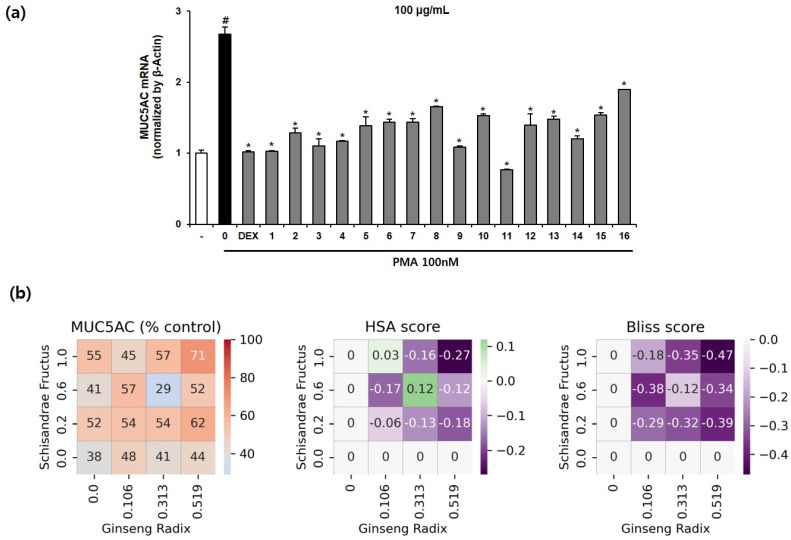
Effects of the 16 multi-herb ratio combinations (MHRCs) on the phorbol 12-myristate 13-acetate (PMA)-induced mRNA expression levels of *MUC5AC* in NCIH292 cells. (**a**) NCI-H292 cells were treated with 10 nM dexamethasone and 16 MHRCs (100 μg/mL) for 1 h before exposure to 100 nM PMA for 24 h. The *MUC5AC* mRNA level was determined using qRT-PCR. The data are expressed as mean ± standard error of the mean (SEM) (n = 2). The numbers on the *x*-axis indicate the codes given for the individual combinations (Table 1). # *p* < 0.05 compared to the untreated group. * *p* < 0.05 compared to the PMA-treated group. DEX, dexamethasone; PMA, phorbol-12-myristate-13-acetate. (**b**) Percent of the test group relative to the negative control, HSA score, and Bliss score for *MUC5AC* mRNA expression. The *x*-axis and *y*-axis represent the dose of drug C or drug D in each cell, and each cell represents one drug combination formulated with the dose of the drug corresponding to the *x*- and *y*-axis. The red color indicates a value higher than that of the positive control.

Phorbol 12-myristate 13-acetate (PMA), one of the substances that increases the expression of genes related to mucus secretion in respiratory epithelial cells, is a protein kinase C (PKC) activator that is known to increase *MUC5AC* expression by inducing an inflammatory response [49]. To investigate the inhibitory effect of 16 MHRCs on PMA-induced *MUC5AC* mRNA expression, 100 μg/mL of MHRCs, a concentration that is non-toxic to NCI-H292 cells (Supplementary Figure S3), was administered as a pretreatment for 1 h, followed by 100 nM PMA for 24 h. Dexamethasone was used as a positive control. The *MUC5AC* mRNA expression level was determined using qPCR. The expression level of *MUC5AC* mRNA was significantly increased by PMA and significantly decreased by the positive control, dexamethasone. Sixteen types of MHRCs significantly decreased *MUC5AC* mRNA expression compared to PMA, and MHRC 11 had a better inhibitory effect than the other 16 combinations (Figure 6a).

Similar to the interaction pattern observed between drugs SF and GR in the RAW264.7 anti-inflammatory model, the expression of *MUC5AC* was not decreased in proportion to the concentration of drugs SF or GR in the expectorant model of NCI-H292. In particular, most combinations had an HSA score lower than 0, indicating that when SF and GR are used simultaneously, the effect of the individual drug is lower than that obtained when combined. In contrast, combination 11 (SF: 0.313, GR: 0.600) and combination 14 (SF: 0.106, GR: 1.000) displayed a greater inhibitory effect than when SF or GR alone was combined; however, the Bliss score was less than 0. This finding indicates that the combined drugs SF and GR may interact to reduce the effect of each other. In summary, combination 11 (SF: 0.313, GR: 0.600) not only had the highest effect among all combinations but also had a higher antitussive expectorant effect than the positive control and a greater effect than the individual drugs (Figure 6b).

### 3.6. DS Analysis for Optimizing the Drug Combination

**Figure 7:**
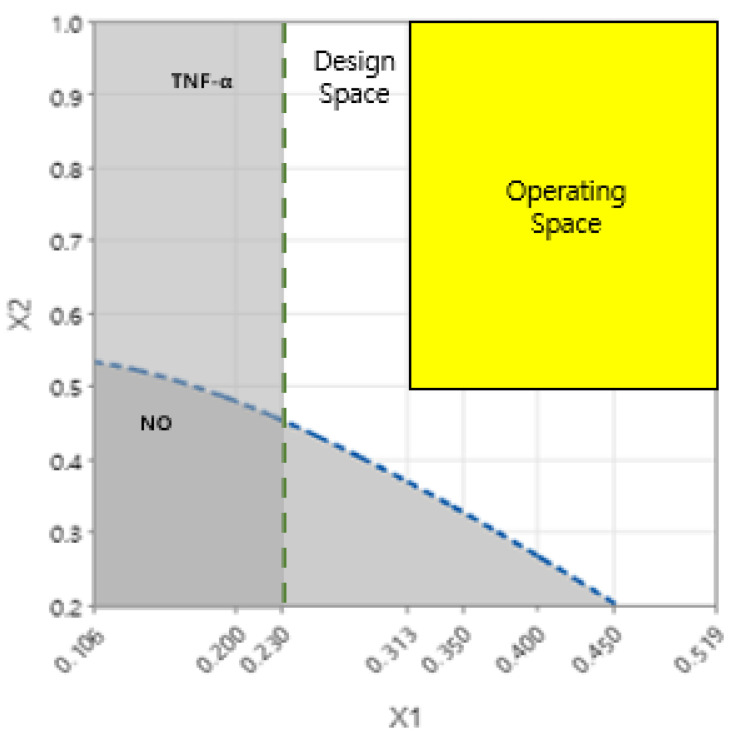
Design space (DS) and operating space (OS) for any combination of factors that satisfy all response criteria. The white-colored area that satisfies the criteria of the target output variable within the level of the input variable is the DS. The yellow-colored area that shows feasible values of factors satisfying all response criteria within the intersection between DS is defined as the OS. The green line and the blue line show upper limits of TNF-α and NO, respectively. The dark color area under the green and blue lines indicates that the quality of the response variable is not guaranteed. The *x*-axis and *y*-axis represent the ratio of factor GR and factor SF in each combination.

In this study, DS was derived using two DOE methods. DS for NO as the response value was derived by applying a central composite face-centered design, which is one of the RSMs. Further, DS for TNF-α, IL-6, IL-1β, and LTB4 as the response value was established using a full factorial design, which is one of the factorial designs. Based on ANOVA, it was statistically significant for NO, TNF-α, IL-6, IL-1β, and LTB4 (*p*-value < 0.05). The parameter for DS was set, as shown in Table 7, and the DS for NO, TNF-α, IL-6, IL-1β, and LTB4 was determined. The OS range of GR and SF was 0.313 to 0.519 and 0.5 to 1.0, respectively. The establishment of the OS was satisfied with the upper limits of all response values. Accordingly, the interaction effect of GR and SF was confirmed to have a positive anti-inflammatory effect (Figure 7).

## 4. Discussion

Post-COVID-19 syndromes are associated with poor quality of life [50], thereby it is necessary to regulate symptoms accompanying post-COVID-19 syndromes. For example, cough is not only one of the most prevalent symptoms of COVID-19 [7] but also a persistent symptom after recovery from COVID-19 [51]. The levels of cytokines including IL-8 and TNF-α, which contribute to the aggravation of COVID-19 infection and represent the morbidity of COVID-19 patients [52], are associated with chronic cough [53,54]. This implies that the regulation of cytokines may contribute to alleviating symptoms after recovery from COVID-19.

A number of reports have indicated that NO possesses potent anti-inflammatory properties. NO is a key signaling molecule which contributes to immune responses triggered by cytokines [55], and functions as a neurotransmitter regulating apoptosis [56] regarded as a pro-inflammatory mediator. In light of these properties, NO inhibitors are considered potential therapeutic options in inflammation associated with the pathogenesis of disorders in the lungs [57].

Dysregulation of MUC5AC, mucin in the mucus layer in bronchioles [58], is considered to be related to diseases in respiratory systems including bronchitis, wheeze, asthma, hay fever, and acute lung injury [59], and interactions between MUC5AC and inflammatory mediators (ERBB1 and IL1RN) are reported to be associated with bronchitis [60].

In the case of NO, it was found that the inhibitory effect appeared only when SF was added at a certain dose or higher. In the case of MUC5AC, the highest performance was shown in drug combination 11 with medium doses of SF and GR, and the synergy decreased when the dose of SF or GR exceeded the medium range. The difference between NO and MUC5AC suggests that the regulation of NO and MUC5AC may be exerted based on the different properties of SF and GR.

Cytokine-signaling pathways including the TNF-α signaling pathway and NF-κB signaling pathway are associated with the production of NO. It was reported that anti-TNF-α reduces the expression of NO synthase in the macrophages [61], and NF-κB activation provokes inducible NO synthase in LPS-induced inflammation [62]. In the case of MUC5AC, it was reported that TNF-α stimulation is correlated with MUC5AC mucin secretion [63], and MUC5AC induced by TNF-α is inhibited by inactivation of NF-κB in NCI-H292 [64]. In our study, combinatory use of EH, SF, PR, and GR showed inhibitory effects on not only inflammatory mediators and production of NO, but also expression of MUC5AC. Moreover, our study found that the targets of EH, SF, and GR are significantly overlapped with genes associated with TNF-α signaling pathway, NF-κB signaling pathway, and calcium signaling pathway. These results suggest that combinatory use of EH, SF, PR and GR decrease the level of NO and MUC5AC by regulating TNF-alpha signaling pathway, NF-κB signaling pathway, and calcium signaling pathway, thereby exert therapeutic effects on inflammation in post-COVID-19. Meanwhile, it was shown that the number of targets in SF is less than those in other herbs. This is due to the fact that compound and target information about SF is limited in the databases used in this study. Nonetheless, this does not simply imply that SF has no connection to the TNF signaling pathway. Actually, SF was reported to inhibit NO, TNF-a, and IL-1b [65]. In addition, there is a possibility that compounds of SF affect the TNF signaling pathway indirectly (via regulation of proteins that interact with other proteins related to the TNF signaling pathway, or action that helps other herbs or metabolites affect pathways), rather than directly acting on proteins related to TNF signaling.

In ELISA, drug combination 4 showed higher inhibitory effects than drug combination 16. However, we note that drug combination 16 also significantly inhibited the cytokines compared to the LPS group. One possible hypothesis is that not only the individual expression of cytokines but also the nonlinear interaction between cytokines, such as the ratio between genes, may have contributed to the change in phenotype (NO inhibition). After the in vitro experiments, therefore, DS analysis was conducted with a quadratic and second-order model to capture the nonlinear interaction between responses.

Our results showed that the drug combination with the highest efficacy is different for the type of markers. In order to comprehensively reflect multiple markers, we input the markers as features to determine the optimal combination in DS analysis. The operating space derived suggests that the corresponding drug combination is optimized to simultaneously regulate multiple factors related to inflammation in the respiratory system.

TCMs which are composed of multiple herbs and complex phytochemical constituents for multi-target treatment, produce synergistic effects [66]. There are many published literatures on the synergistic effects of TCMs or their components. However, few study has conducted by both experimental and network pharmacological method with synergistic efficacy. In the first example, a mixture of ginkgolide A and B, which are extracted from standardized *Ginkgo biloba*, has shown a synergistic effect through in vitro platelet aggregation test with isobol method, in which doses of one constituent are represented along one axis and doses of a second one are displayed on the second axis. The isobol curve includes combinations of doses for a specific efficacy level. If the constituents do not interact, the curve will be a straight line [67]. Han et al. demonstrated synergistic anti-candidal effect of epigallocatchin-*O*-gallate (EGCG) with Amp B (amphotericin B) showed in animal model. The mean survival time (MST) from the combination-administrated mice group was much longer than that from the Amp B group [68]. Chen et al. reported that Qixue shuangbu prescription is used for treating of chronic heart failure and produced a synergistic effects based on a network pharmacology that integrated metabolic strategy and network pharmacological tool without any experimental work [69]. A study by Zhao et al. has shown that the combination of main active compounds, dihydromyricetin (DMY) and myricitrin (MYT) of vine extract on B16F10 cells was more effective than each active compound when the combination in different range was analyzed by in vitro experiment such as proliferation assay, enzyme assay, cellular fluorescence staining and flow cytometry. The combination index (CI) calculated the synergistic effect and network pharmacology was used to discuss its mechanism [70].

As compared with other synergy research, we designed and tried to demonstrate the experiments more closely related to respiratory disease. Additionally, we grafted network pharmacological analysis and various statistical methods onto the in vitro data. We tried to evaluate the damage to the respiratory tract by using NCI-H292 cells even though our milieu is different from SARS-CoV-2. Nonetheless, the pathological inflammation in patients with the infection has similarities to our condition induced by LPS in RAW264.7 macrophage cells.

Noteworthy in this study was the systemically used network pharmacological analysis with experimental data. In the network pharmacological methods, we applied robust (also generally preferred) criteria for potential bioactive compounds to minimize the false-positive results, and there is possibility that some potential therapeutic compounds would be omitted. However, there were several limitations to this research. Firstly, more detailed combinations should be quantitatively tested for synergistic effect. Secondly, synergistic effectiveness of our combinations was evaluated only with in vitro experiments, without animal experiment. Thirdly, metabolism on the drug combination should consider by network pharmacological intervention. Lastly, numerous unidentified ingredients of GR and SF as well as EH and PR were still included in the combinations.

## 5. Conclusions

We investigated the potential mechanisms of drug combinations comprising EH, PR, GR, and SF; validated the anti-inflammatory effects of the drugs; and determined the optimal doses of the drug combinations. The associations of herbs with tissues (such as bronchial epithelial cells and lungs) and pathways (such as TNF, NF-κB, and calcium signaling pathways) were discovered by constructing an herb-compound-target network. The anti-inflammatory effects of the drug combinations were demonstrated by their inhibition of the production of NO and inflammatory mediators, including TNF-α, IL-6, IL-1β, and leukotriene B4, in the RAW264.7 cell line treated with LPS. In addition, the drug combination inhibited PMA-induced *MUC5AC* mRNA expression in NCI-H292 cells. The optimal herbal medicine combinations were determined by performing a DS analysis using DOE and synergy score calculation. Consequently, a mitigating effect on inflammation in COVID-19 was confirmed through a combination study of herbal preparations.

## Figures and Tables

**Table 1:** Combined doses of the herbal extract used to confirm the synergistic effect.

	EH (g)	PR (g)	SF (g)	GR (g)
**1**	0.273	0.263	0.000	0.000
**2**	0.273	0.263	0.106	0.000
**3**	0.273	0.263	0.313	0.000
**4**	0.273	0.263	0.519	0.000
**5**	0.273	0.263	0.000	0.200
**6**	0.273	0.263	0.106	0.200
**7**	0.273	0.263	0.313	0.200
**8**	0.273	0.263	0.519	0.200
**9**	0.273	0.263	0.000	0.600
**10**	0.273	0.263	0.106	0.600
**11**	0.273	0.263	0.313	0.600
**12**	0.273	0.263	0.519	0.600
**13**	0.273	0.263	0.000	1.000
**14**	0.273	0.263	0.106	1.000
**15**	0.273	0.263	0.313	1.000
**16**	0.273	0.263	0.519	1.000

**Table 2:** Primer Sequences.

Gene	Sense Primer Sequence (5′-3′)	Antisense Primer Sequence (5′-3′)
MUC5AC	TCCACCATATACCGCCACAGA	TGGACGGACAGTCACTGTCAAC
β-actin	AGGAGAAGCTGTGCTACGTC	GGATGTCCACGTCACACTTC

Abbreviations: MUC5AC, Mucin 5AC.

**Table 3:** Enrichment analysis of the impact of each drug on COVID-19-related genes.

Database	Drug	Overlap	*p*-Value (Adjusted)	Odds Ratio	Combined Score
CTD database	Drug combination	11/37	5.72 × 10^−5^	6.33	72.02
	Ephedrae Herba	9/37	0.00142	5.02	41.00
	Platycodonis Radix	7/37	4.37 × 10^−4^	7.80	72.90
	Ginseng Radix	8/37	5.81 × 10^−5^	9.01	102.38
	Schisandrae Fructus	0/37	-	-	-
KEGG	Drug combination	37/232	8.11 × 10^−12^	4.47	119.04
	Ephedrae Herba	35/232	4.64 × 10^−11^	4.37	108.57
	Platycodonis Radix	20/232	1.14 × 10^−7^	4.92	84.43
	Ginseng Radix	20/232	1.61 × 10^−7^	4.79	80.20
	Schisandrae Fructus	1/232	0.24	4.07	6.05

**Table 4:** Enrichment analysis of the impact of each drug on the tissue types related to COVID-19.

Tissue	Drug	Overlap	*p*-Value (Adjusted)	Odds Ratio	Combined Score
Bronchial epithelial cells	Drug combination	30/280	5.67 × 10^−5^	2.79	35.73
	Ephedrae Herba	30/280	2.40 × 10^−5^	2.93	39.92
	Platycodonis Radix	9/280	0.45	1.68	3.84
	Ginseng Radix	11/280	0.26	2.02	7.32
	Schisandrae Fructus	1/280	0.40	3.36	4.44
Lung	Drug combination	22/299	0.047	1.83	8.78
	Ephedrae Herba	21/299	0.063	1.82	8.35
	Platycodonis Radix	12/299	0.13	2.13	8.87
	Ginseng Radix	11/299	0.28	1.89	6.07
	Schisandrae Fructus	0/299	-	-	-

**Table 5:** Enrichment analysis results of the impact of each drug on the pathways related to TNF-α, IL-1β, and BLT1.

Pathway	Drug	Overlap	*p*-Value (Adjusted)	Odds Ratio	Combined Score
TNF signaling pathway	Drug combination	36/112	2.68 × 10^−21^	11.21	560.76
	Ephedrae Herba	31/112	4.54 × 10^−17^	9.43	373.21
	Platycodonis Radix	20/112	8.17 × 10^−13^	11.40	348.36
	Ginseng Radix	20/112	1.12 × 10^−12^	11.10	333.83
	Schisandrae Fructus	0/112	-	-	-
NF-κB signaling pathway	Drug combination	23/104	1.13 × 10^−10^	6.61	157.46
	Ephedrae Herba	21/104	2.05 × 10^−9^	6.15	128.27
	Platycodonis Radix	9/104	3.69 × 10^−4^	4.83	40.93
	Ginseng Radix	11/104	1.76 × 10^−5^	5.90	69.33
	Schisandrae Fructus	0/104	-	-	-
Calcium signaling pathway	Drug combination	66/240	7.96 × 10^−34^	9.28	743.30
	Ephedrae Herba	66/240	7.03 × 10^−35^	9.74	805.21
	Platycodonis Radix	37/240	5.74 × 10^−21^	9.96	500.62
	Ginseng Radix	38/240	1.55 × 10^−21^	10.02	517.97
	Schisandrae Fructus	1/240	0.25	3.93	5.72

**Table 6:** Bioactive compounds predicted to interact with the proteins associated with TNF-α, NF-κB, and calcium signaling pathways.

Drug	Compounds
Ephedrae Herba	hexanoic acid, carvacrol, pentadecanoic acid, tricin, ethyl acetate, cinnamaldehyde, tetradecanoic acid, guaiazulene, ethanol, diethyl sulfate, p-cresol, dibutyl phthalate, apigenin, gamma-butyrolactone, safranal, 2,3,5,6-tetramethylpyrazine, pseudoephedrine, farnesol, palmitic acid, quercetin, nonanoic acid, kaempferol, lauric acid, decanoic acid, thymol, styrene, hexanal, phenanthrene, toluene, ephedrine
Platycodonis Radix	palmitic acid, luteolin, 2-cyclopenten-1-one, acacetin
Ginseng Radix	Stigmasterol, paeonol, protopanaxatriol, pentadecanoic acid, palmitic acid, octane, kaempferol, beta-elemene, 6-methoxyluteolin, beta-sitosterol, dibutyl phthalate, (20s)-protopanaxatriol
Schisandrae Fructus	dibutyl phthalate

**Table 7:** Parameter of the output variables for DS.

Responses	Target	Upper Limit	Criteria for Setting the Upper Limit
Nitric Oxide	Minimum	18.73	10% improvement compared to LPS
TNF-α	Minimum	6682.51	30% improvement compared to LPS
IL-6	Minimum	1777.42	50% improvement compared to LPS
IL-1β	Minimum	9.06	50% improvement compared to LPS
LTB4	Minimum	4.47	50% improvement compared to LPS

## Data Availability

All relevant data of this study are presented. Additional data will be provided upon request.

## Supplementary Materials

The following supporting information can be downloaded at: https://www.mdpi.com/article/10.3390/healthcare11010143/s1, Figure S1: A pathway for coronavirus diseases highlighting the potential targets of the drug combination; Figure S2: Viability of the drug combinations in Raw264.7 cells. Figure S3: Viability of the drug combinations in NCI-H29cells.
