# Supplementary material for: Network Pharmacological Analysis on the Herbal Combinations for Mitigating Inflammation in Respiratory Tracts and Experimental Evaluation
Source: Healthcare (Basel). 2023 Jan 3;11(1):143. doi: 10.3390/healthcare11010143 (PMC9819683; doi:10.3390/healthcare11010143)
Supplement: Supplementary file 1 [file healthcare-11-00143-s001.zip › healthcare-2070997-supplementary.pdf]

healthcare

MDPI

**Network pharmacological analysis on the herbal combinations for mitigating inflammation in respiratory tracts and experimental evaluation**

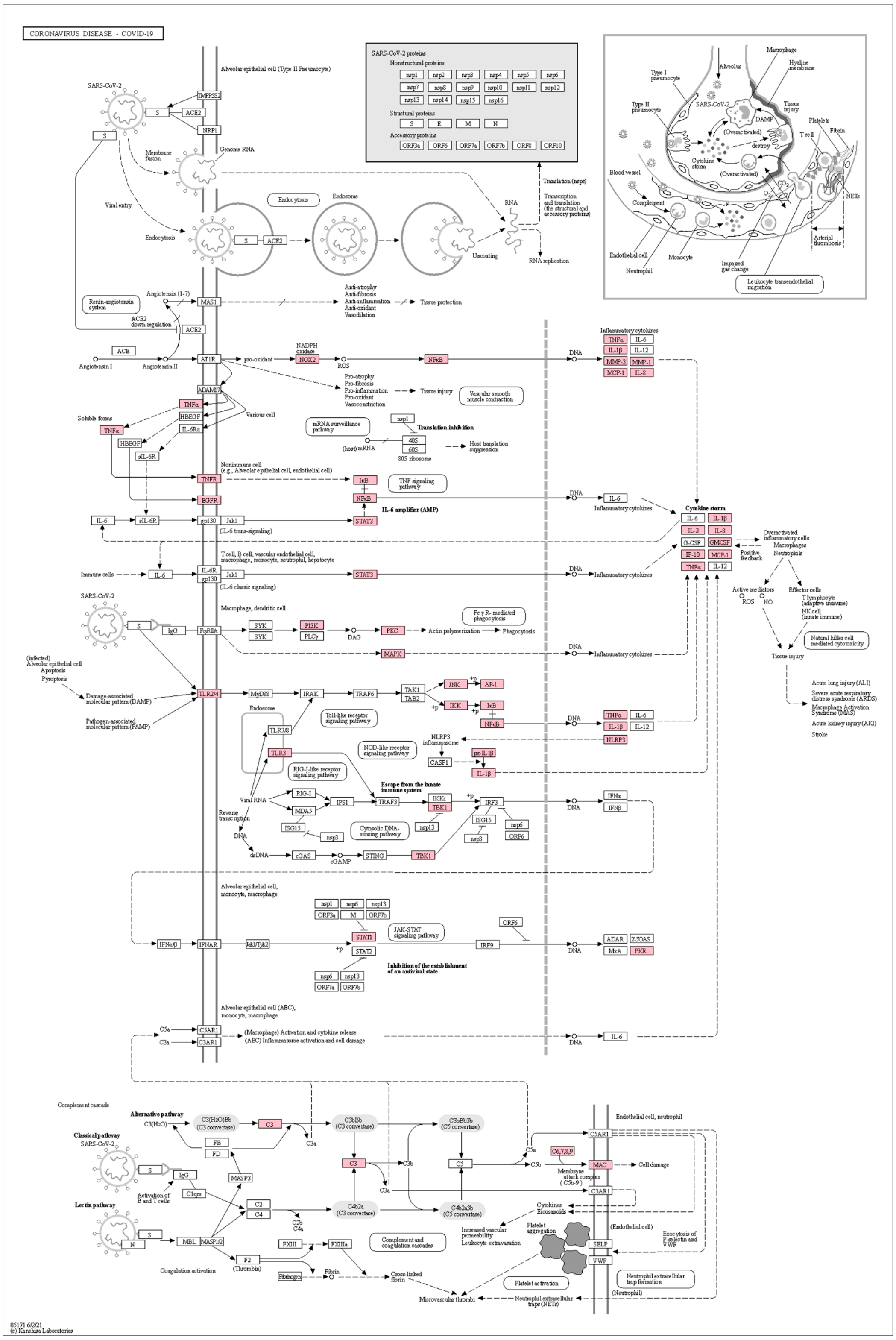


**Supplementary Figure S1**. A pathway for coronavirus diseases highlighting the potential targets of the drug combination. Boxes and arrows between them indicate the proteins that consist of the pathway and interactions between the proteins, respectively. Pink-colored boxes indicate the targets of the drug combination. The graph image was generated by a KEGG mapper.

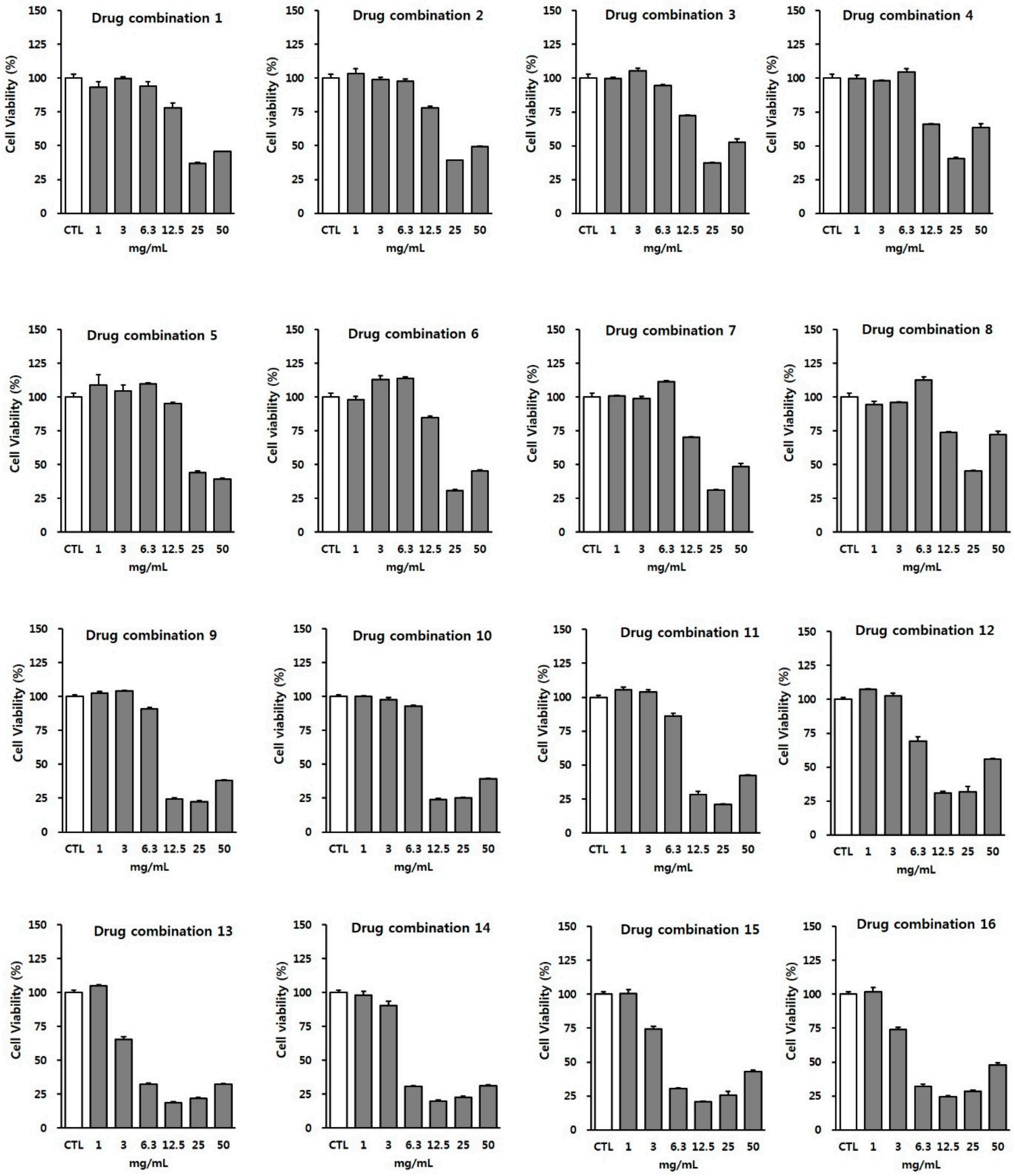


**Supplementary Figure S2.** Viability of the drug combinations in RAW 264.7cells. RAW 264.7 cells were treated with the drug combinations for 24h. Cell viability was determined using EZ-Cytox assay.

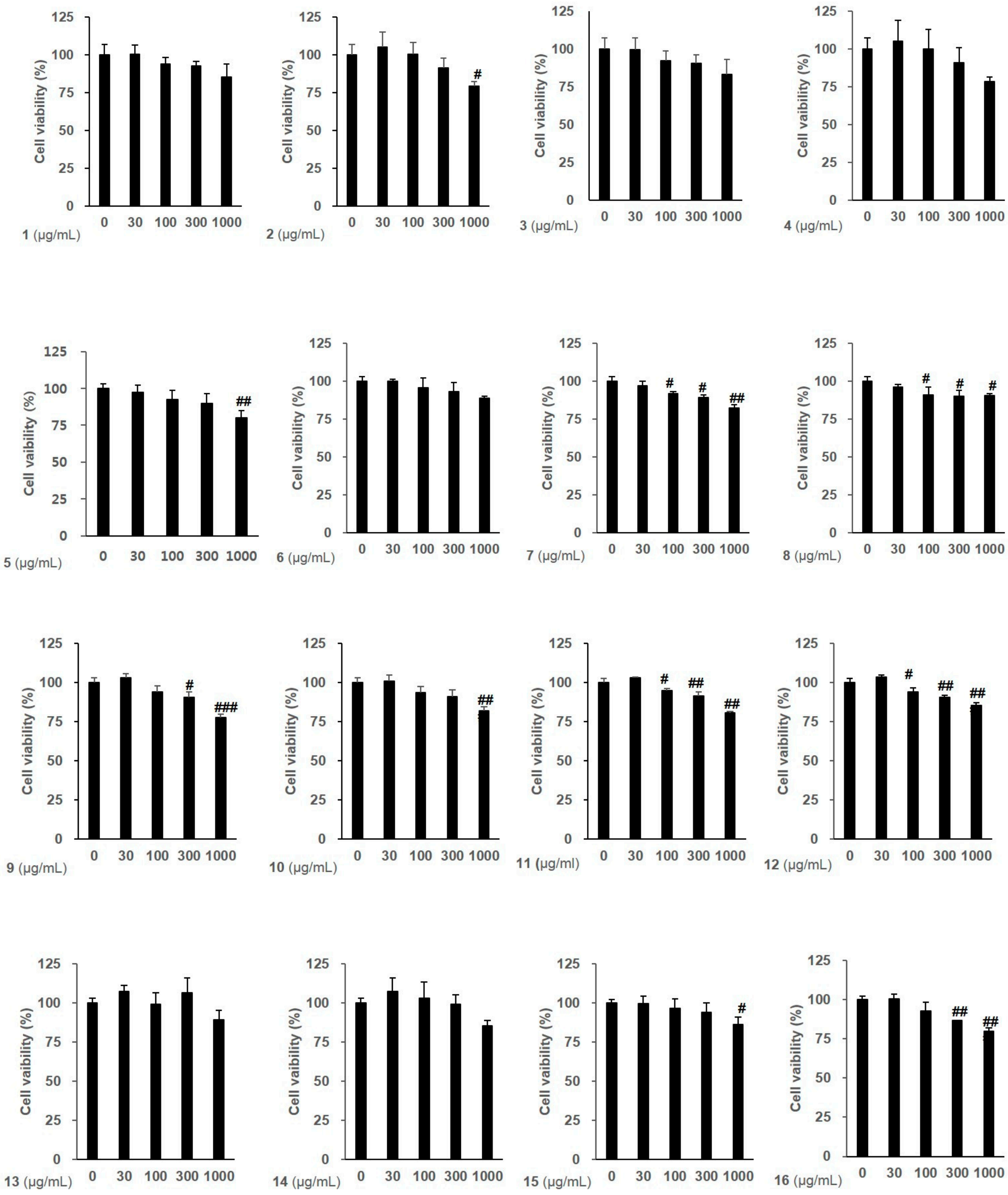


**Supplementary Figure S3. Viability of the drug combinations in NCI-H292 cells.** NCI-H292 cells were treated with various concentrations of the drug combinations for 24 h. Cell viability was determined using the Ez-Cytox kit. The data are presented as mean ± SEM (n = 3). # $p < 0.05$, ## $p < 0.01$, and ### $p < 0.001$ compared to non-treated group.
